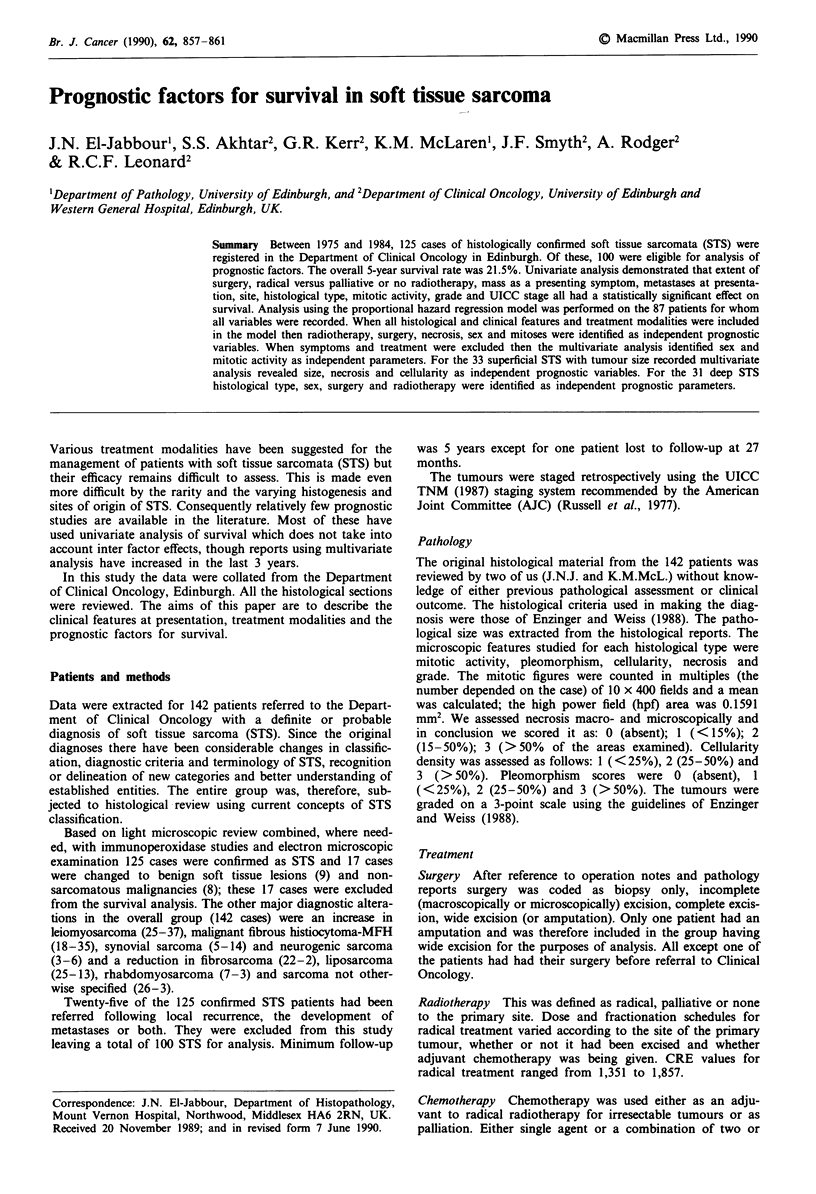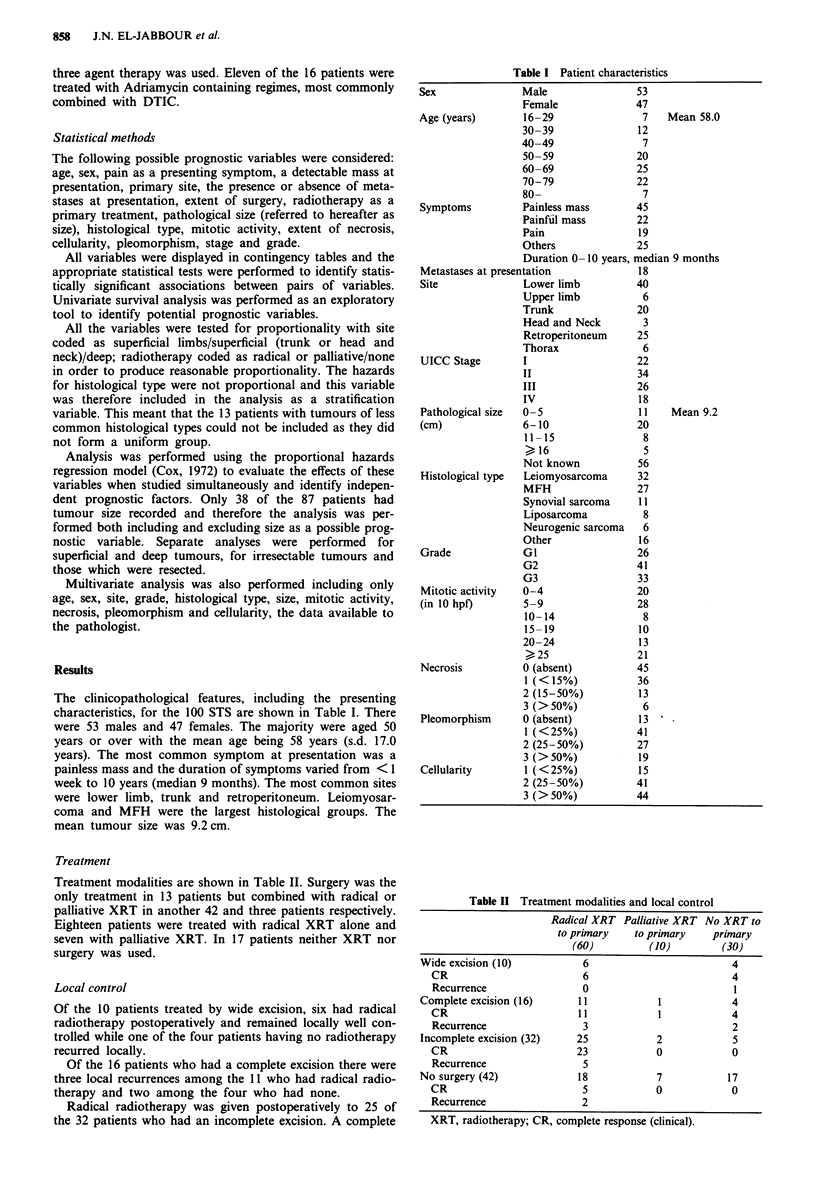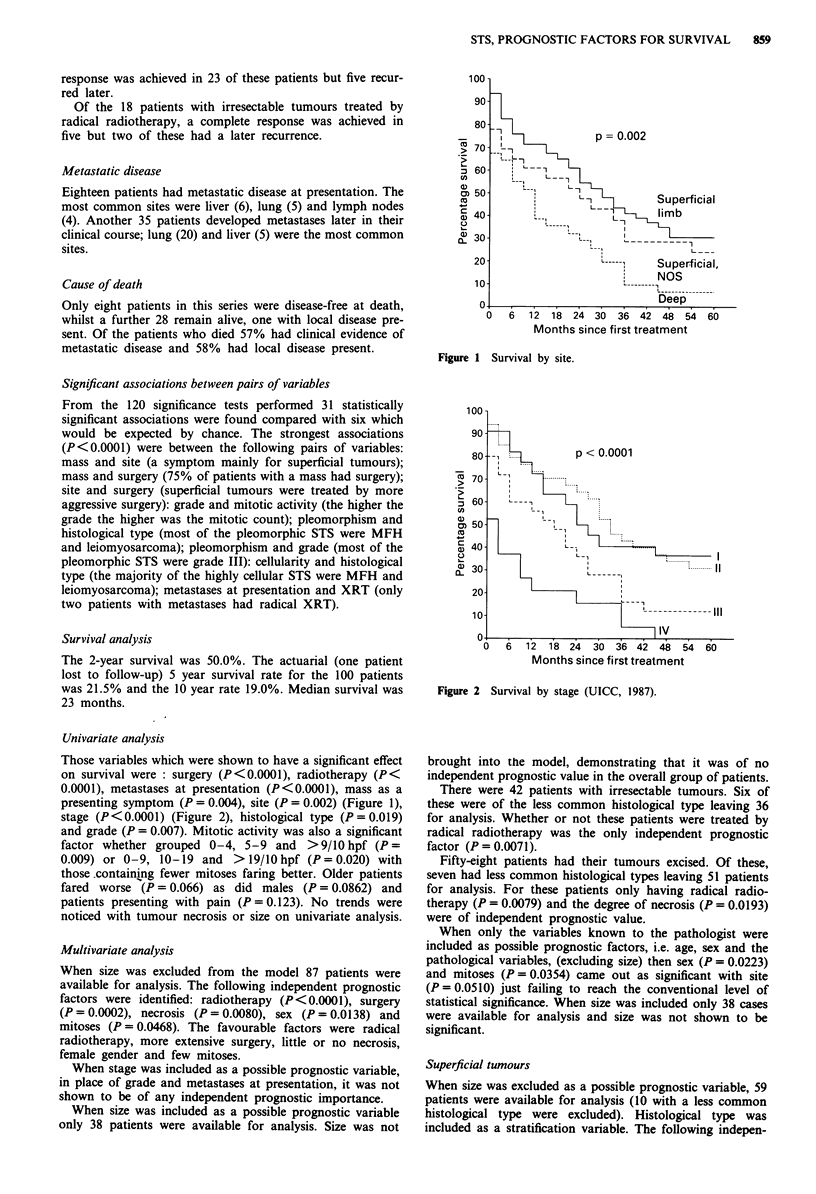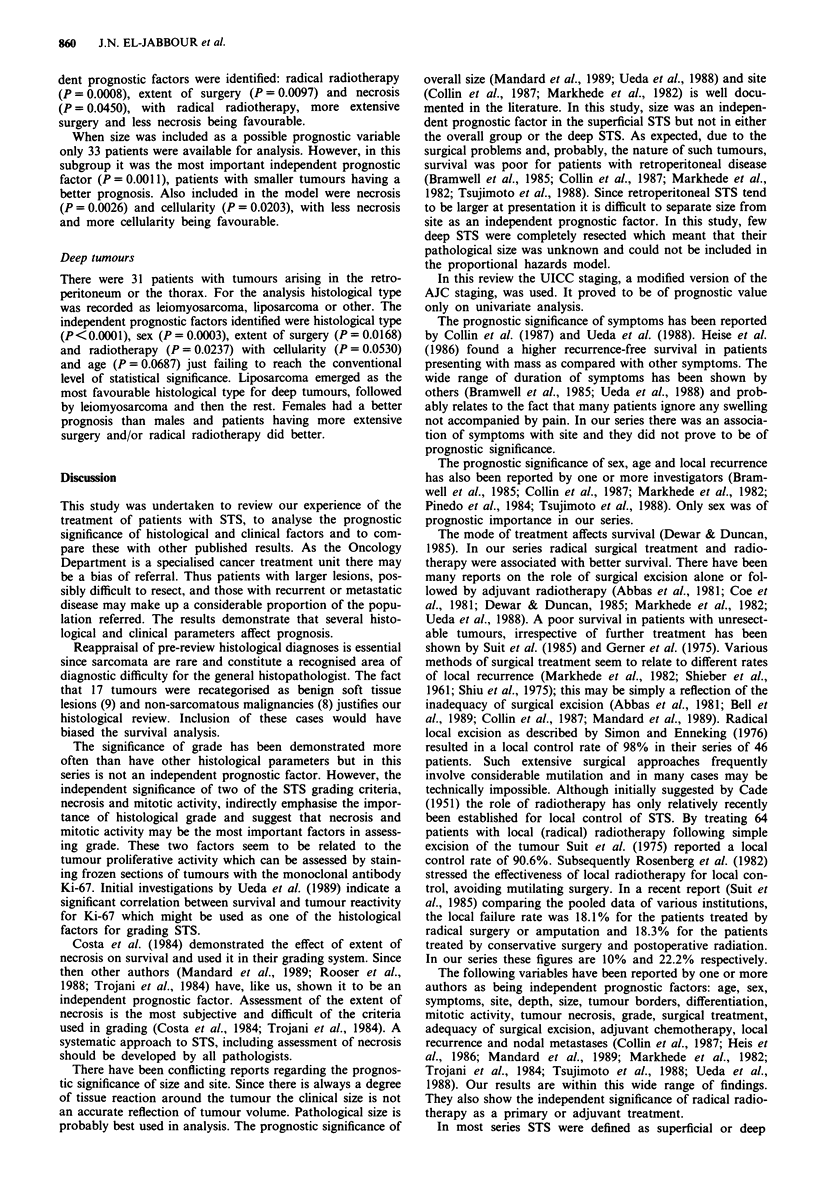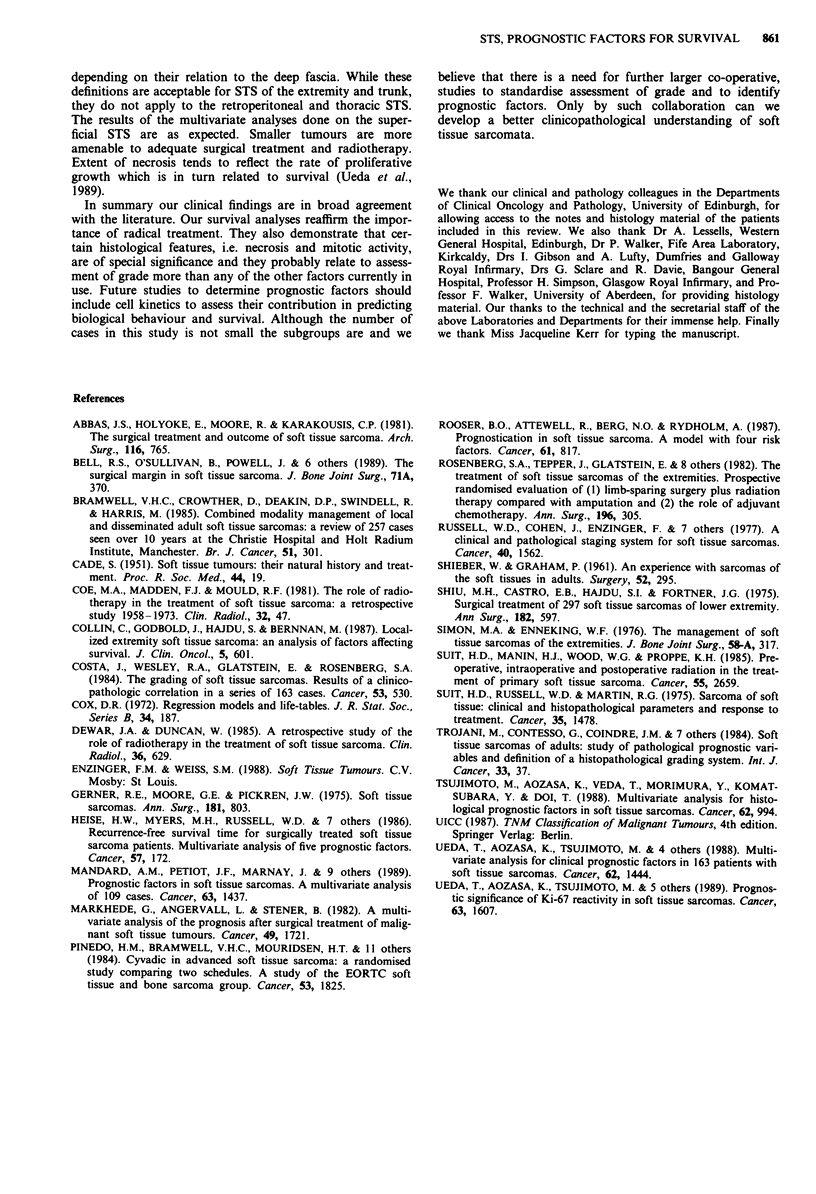# Prognostic factors for survival in soft tissue sarcoma.

**DOI:** 10.1038/bjc.1990.394

**Published:** 1990-11

**Authors:** J. N. el-Jabbour, S. S. Akhtar, G. R. Kerr, K. M. McLaren, J. F. Smyth, A. Rodger, R. C. Leonard

**Affiliations:** Department of Pathology, University of Edinburgh, UK.

## Abstract

Between 1975 and 1984, 125 cases of histologically confirmed soft tissue sarcomata (STS) were registered in the Department of Clinical Oncology in Edinburgh. Of these, 100 were eligible for analysis of prognostic factors. The overall 5-year survival rate was 21.5%. Univariate analysis demonstrated that extent of surgery, radical versus palliative or no radiotherapy, mass as a presenting symptom, metastases at presentation, site, histological type, mitotic activity, grade and UICC stage all had a statistically significant effect on survival. Analysis using the proportional hazard regression model was performed on the 87 patients for whom all variables were recorded. When all histological and clinical features and treatment modalities were included in the model then radiotherapy, surgery, necrosis, sex and mitoses were identified as independent prognostic variables. When symptoms and treatment were excluded then the multivariate analysis identified sex and mitotic activity as independent parameters. For the 33 superficial STS with tumour size recorded multivariate analysis revealed size, necrosis and cellularity as independent prognostic variables. For the 31 deep STS histological type, sex, surgery and radiotherapy were identified as independent prognostic parameters.


Br. J. Cancer (1990), 62, 857-861
© Macmillan Press Ltd., 1990

### Prognostic factors for survival in soft tissue sarcoma

J.N. El-Jabbour[1], S.S. Akhtar[2], G.R. Kerr[2], K.M. McLaren[1], J.F. Smyth[2], A. Rodger[2] & R.C.F. Leonard[2]

[1]*Department of Pathology, University of Edinburgh, and* [2]*Department of Clinical Oncology, University of Edinburgh and Western General Hospital, Edinburgh, UK.*

**Summary** Between 1975 and 1984, 125 cases of histologically confirmed soft tissue sarcomata (STS) were registered in the Department of Clinical Oncology in Edinburgh. Of these, 100 were eligible for analysis of prognostic factors. The overall 5-year survival rate was 21.5%. Univariate analysis demonstrated that extent of surgery, radical versus palliative or no radiotherapy, mass as a presenting symptom, metastases at presentation, site, histological type, mitotic activity, grade and UICC stage all had a statistically significant effect on survival. Analysis using the proportional hazard regression model was performed on the 87 patients for whom all variables were recorded. When all histological and clinical features and treatment modalities were included in the model then radiotherapy, surgery, necrosis, sex and mitoses were identified as independent prognostic variables. When symptoms and treatment were excluded then the multivariate analysis identified sex and mitotic activity as independent parameters. For the 33 superficial STS with tumour size recorded multivariate analysis revealed size, necrosis and cellularity as independent prognostic variables. For the 31 deep STS histological type, sex, surgery and radiotherapy were identified as independent prognostic parameters.

Various treatment modalities have been suggested for the management of patients with soft tissue sarcomata (STS) but their efficacy remains difficult to assess. This is made even more difficult by the rarity and the varying histogenesis and sites of origin of STS. Consequently relatively few prognostic studies are available in the literature. Most of these have used univariate analysis of survival which does not take into account inter factor effects, though reports using multivariate analysis have increased in the last 3 years.

In this study the data were collated from the Department of Clinical Oncology, Edinburgh. All the histological sections were reviewed. The aims of this paper are to describe the clinical features at presentation, treatment modalities and the prognostic factors for survival.

### Patients and methods

Data were extracted for 142 patients referred to the Department of Clinical Oncology with a definite or probable diagnosis of soft tissue sarcoma (STS). Since the original diagnoses there have been considerable changes in classification, diagnostic criteria and terminology of STS, recognition or delineation of new categories and better understanding of established entities. The entire group was, therefore, subjected to histological review using current concepts of STS classification.

Based on light microscopic review combined, where needed, with immunoperoxidase studies and electron microscopic examination 125 cases were confirmed as STS and 17 cases were changed to benign soft tissue lesions (9) and non-sarcomatous malignancies (8); these 17 cases were excluded from the survival analysis. The other major diagnostic alterations in the overall group (142 cases) were an increase in leiomyosarcoma (25-37), malignant fibrous histiocytoma-MFH (18-35), synovial sarcoma (5-14) and neurogenic sarcoma (3-6) and a reduction in fibrosarcoma (22-2), liposarcoma (25-13), rhabdomyosarcoma (7-3) and sarcoma not otherwise specified (26-3).

Twenty-five of the 125 confirmed STS patients had been referred following local recurrence, the development of metastases or both. They were excluded from this study leaving a total of 100 STS for analysis. Minimum follow-up was 5 years except for one patient lost to follow-up at 27 months.

The tumours were staged retrospectively using the UICC TNM (1987) staging system recommended by the American Joint Committee (AJC) (Russell *et al.*, 1977).

### Pathology

The original histological material from the 142 patients was reviewed by two of us (J.N.J. and K.M.McL.) without knowledge of either previous pathological assessment or clinical outcome. The histological criteria used in making the diagnosis were those of Enzinger and Weiss (1988). The pathological size was extracted from the histological reports. The microscopic features studied for each histological type were mitotic activity, pleomorphism, cellularity, necrosis and grade. The mitotic figures were counted in multiples (the number depended on the case) of $10 \times 400$ fields and a mean was calculated; the high power field (hpf) area was 0.1591 $mm^2$. We assessed necrosis macro- and microscopically and in conclusion we scored it as: 0 (absent); 1 (<15%); 2 (15-50%); 3 (>50% of the areas examined). Cellularity density was assessed as follows: 1 (<25%), 2 (25-50%) and 3 (>50%). Pleomorphism scores were 0 (absent), 1 (<25%), 2 (25-50%) and 3 (>50%). The tumours were graded on a 3-point scale using the guidelines of Enzinger and Weiss (1988).

### Treatment

*Surgery* After reference to operation notes and pathology reports surgery was coded as biopsy only, incomplete (macroscopically or microscopically) excision, complete excision, wide excision (or amputation). Only one patient had an amputation and was therefore included in the group having wide excision for the purposes of analysis. All except one of the patients had had their surgery before referral to Clinical Oncology.

*Radiotherapy* This was defined as radical, palliative or none to the primary site. Dose and fractionation schedules for radical treatment varied according to the site of the primary tumour, whether or not it had been excised and whether adjuvant chemotherapy was being given. CRE values for radical treatment ranged from 1,351 to 1,857.

*Chemotherapy* Chemotherapy was used either as an adjuvant to radical radiotherapy for irresectable tumours or as palliation. Either single agent or a combination of two or

Correspondence: J.N. El-Jabbour, Department of Histopathology, Mount Vernon Hospital, Northwood, Middlesex HA6 2RN, UK.
Received 20 November 1989; and in revised form 7 June 1990.

three agent therapy was used. Eleven of the 16 patients were treated with Adriamycin containing regimes, most commonly combined with DTIC.

*Statistical methods*

The following possible prognostic variables were considered: age, sex, pain as a presenting symptom, a detectable mass at presentation, primary site, the presence or absence of metastases at presentation, extent of surgery, radiotherapy as a primary treatment, pathological size (referred to hereafter as size), histological type, mitotic activity, extent of necrosis, cellularity, pleomorphism, stage and grade.

All variables were displayed in contingency tables and the appropriate statistical tests were performed to identify statistically significant associations between pairs of variables. Univariate survival analysis was performed as an exploratory tool to identify potential prognostic variables.

All the variables were tested for proportionality with site coded as superficial limbs/superficial (trunk or head and neck)/deep; radiotherapy coded as radical or palliative/none in order to produce reasonable proportionality. The hazards for histological type were not proportional and this variable was therefore included in the analysis as a stratification variable. This meant that the 13 patients with tumours of less common histological types could not be included as they did not form a uniform group.

Analysis was performed using the proportional hazards regression model (Cox, 1972) to evaluate the effects of these variables when studied simultaneously and identify independent prognostic factors. Only 38 of the 87 patients had tumour size recorded and therefore the analysis was performed both including and excluding size as a possible prognostic variable. Separate analyses were performed for superficial and deep tumours, for irresectable tumours and those which were resected.

Multivariate analysis was also performed including only age, sex, site, grade, histological type, size, mitotic activity, necrosis, pleomorphism and cellularity, the data available to the pathologist.

### Results

The clinicopathological features, including the presenting characteristics, for the 100 STS are shown in Table I. There were 53 males and 47 females. The majority were aged 50 years or over with the mean age being 58 years (s.d. 17.0 years). The most common symptom at presentation was a painless mass and the duration of symptoms varied from < 1 week to 10 years (median 9 months). The most common sites were lower limb, trunk and retroperitoneum. Leiomyosarcoma and MFH were the largest histological groups. The mean tumour size was 9.2 cm.

**Table I** Patient characteristics

| | | | |
|---|---|---|---|
| Sex | Male | 53 | |
| | Female | 47 | |
| Age (years) | 16–29 | 7 | Mean 58.0 |
| | 30–39 | 12 | |
| | 40–49 | 7 | |
| | 50–59 | 20 | |
| | 60–69 | 25 | |
| | 70–79 | 22 | |
| | 80– | 7 | |
| Symptoms | Painless mass | 45 | |
| | Painful mass | 22 | |
| | Pain | 19 | |
| | Others | 25 | |
| | Duration 0–10 years, median 9 months | | |
| Metastases at presentation | | 18 | |
| Site | Lower limb | 40 | |
| | Upper limb | 6 | |
| | Trunk | 20 | |
| | Head and Neck | 3 | |
| | Retroperitoneum | 25 | |
| | Thorax | 6 | |
| UICC Stage | I | 22 | |
| | II | 34 | |
| | III | 26 | |
| | IV | 18 | |
| Pathological size (cm) | 0–5 | 11 | Mean 9.2 |
| | 6–10 | 20 | |
| | 11–15 | 8 | |
| | ⩾16 | 5 | |
| | Not known | 56 | |
| Histological type | Leiomyosarcoma | 32 | |
| | MFH | 27 | |
| | Synovial sarcoma | 11 | |
| | Liposarcoma | 8 | |
| | Neurogenic sarcoma | 6 | |
| | Other | 16 | |
| Grade | G1 | 26 | |
| | G2 | 41 | |
| | G3 | 33 | |
| Mitotic activity (in 10 hpf) | 0–4 | 20 | |
| | 5–9 | 28 | |
| | 10–14 | 8 | |
| | 15–19 | 10 | |
| | 20–24 | 13 | |
| | ⩾25 | 21 | |
| Necrosis | 0 (absent) | 45 | |
| | 1 (<15%) | 36 | |
| | 2 (15–50%) | 13 | |
| | 3 (>50%) | 6 | |
| Pleomorphism | 0 (absent) | 13 | |
| | 1 (<25%) | 41 | |
| | 2 (25–50%) | 27 | |
| | 3 (>50%) | 19 | |
| Cellularity | 1 (<25%) | 15 | |
| | 2 (25–50%) | 41 | |
| | 3 (>50%) | 44 | |

*Treatment*

Treatment modalities are shown in Table II. Surgery was the only treatment in 13 patients but combined with radical or palliative XRT in another 42 and three patients respectively. Eighteen patients were treated with radical XRT alone and seven with palliative XRT. In 17 patients neither XRT nor surgery was used.

*Local control*

Of the 10 patients treated by wide excision, six had radical radiotherapy postoperatively and remained locally well controlled while one of the four patients having no radiotherapy recurred locally.

Of the 16 patients who had a complete excision there were three local recurrences among the 11 who had radical radiotherapy and two among the four who had none.

Radical radiotherapy was given postoperatively to 25 of the 32 patients who had an incomplete excision. A complete

**Table II** Treatment modalities and local control

| | *Radical XRT to primary (60)* | *Palliative XRT to primary (10)* | *No XRT to primary (30)* |
|---|---|---|---|
| Wide excision (10) | 6 | | 4 |
| CR | 6 | | 4 |
| Recurrence | 0 | | 1 |
| Complete excision (16) | 11 | 1 | 4 |
| CR | 11 | 1 | 4 |
| Recurrence | 3 | | 2 |
| Incomplete excision (32) | 25 | 2 | 5 |
| CR | 23 | 0 | 0 |
| Recurrence | 5 | | |
| No surgery (42) | 18 | 7 | 17 |
| CR | 5 | 0 | 0 |
| Recurrence | 2 | | |

XRT, radiotherapy; CR, complete response (clinical).

response was achieved in 23 of these patients but five recurred later.

Of the 18 patients with irresectable tumours treated by radical radiotherapy, a complete response was achieved in five but two of these had a later recurrence.

*Metastatic disease*

Eighteen patients had metastatic disease at presentation. The most common sites were liver (6), lung (5) and lymph nodes (4). Another 35 patients developed metastases later in their clinical course; lung (20) and liver (5) were the most common sites.

*Cause of death*

Only eight patients in this series were disease-free at death, whilst a further 28 remain alive, one with local disease present. Of the patients who died 57% had clinical evidence of metastatic disease and 58% had local disease present.

*Significant associations between pairs of variables*

From the 120 significance tests performed 31 statistically significant associations were found compared with six which would be expected by chance. The strongest associations ($P<0.0001$) were between the following pairs of variables: mass and site (a symptom mainly for superficial tumours); mass and surgery (75% of patients with a mass had surgery); site and surgery (superficial tumours were treated by more aggressive surgery): grade and mitotic activity (the higher the grade the higher was the mitotic count); pleomorphism and histological type (most of the pleomorphic STS were MFH and leiomyosarcoma); pleomorphism and grade (most of the pleomorphic STS were grade III): cellularity and histological type (the majority of the highly cellular STS were MFH and leiomyosarcoma); metastases at presentation and XRT (only two patients with metastases had radical XRT).

*Survival analysis*

The 2-year survival was 50.0%. The actuarial (one patient lost to follow-up) 5 year survival rate for the 100 patients was 21.5% and the 10 year rate 19.0%. Median survival was 23 months.

*Univariate analysis*

Those variables which were shown to have a significant effect on survival were : surgery ($P<0.0001$), radiotherapy ($P<0.0001$), metastases at presentation ($P<0.0001$), mass as a presenting symptom ($P=0.004$), site ($P=0.002$) (Figure 1), stage ($P<0.0001$) (Figure 2), histological type ($P=0.019$) and grade ($P=0.007$). Mitotic activity was also a significant factor whether grouped 0–4, 5–9 and >9/10 hpf ($P=0.009$) or 0–9, 10–19 and >19/10 hpf ($P=0.020$) with those containing fewer mitoses faring better. Older patients fared worse ($P=0.066$) as did males ($P=0.0862$) and patients presenting with pain ($P=0.123$). No trends were noticed with tumour necrosis or size on univariate analysis.

*Multivariate analysis*

When size was excluded from the model 87 patients were available for analysis. The following independent prognostic factors were identified: radiotherapy ($P<0.0001$), surgery ($P=0.0002$), necrosis ($P=0.0080$), sex ($P=0.0138$) and mitoses ($P=0.0468$). The favourable factors were radical radiotherapy, more extensive surgery, little or no necrosis, female gender and few mitoses.

When stage was included as a possible prognostic variable, in place of grade and metastases at presentation, it was not shown to be of any independent prognostic importance.

When size was included as a possible prognostic variable only 38 patients were available for analysis. Size was not

**Figure 1** Survival by site.

**Figure 2** Survival by stage (UICC, 1987).

brought into the model, demonstrating that it was of no independent prognostic value in the overall group of patients.

There were 42 patients with irresectable tumours. Six of these were of the less common histological type leaving 36 for analysis. Whether or not these patients were treated by radical radiotherapy was the only independent prognostic factor ($P=0.0071$).

Fifty-eight patients had their tumours excised. Of these, seven had less common histological types leaving 51 patients for analysis. For these patients only having radical radiotherapy ($P=0.0079$) and the degree of necrosis ($P=0.0193$) were of independent prognostic value.

When only the variables known to the pathologist were included as possible prognostic factors, i.e. age, sex and the pathological variables, (excluding size) then sex ($P=0.0223$) and mitoses ($P=0.0354$) came out as significant with site ($P=0.0510$) just failing to reach the conventional level of statistical significance. When size was included only 38 cases were available for analysis and size was not shown to be significant.

*Superficial tumours*

When size was excluded as a possible prognostic variable, 59 patients were available for analysis (10 with a less common histological type were excluded). Histological type was included as a stratification variable. The following independent prognostic factors were identified: radical radiotherapy ($P = 0.0008$), extent of surgery ($P = 0.0097$) and necrosis ($P = 0.0450$), with radical radiotherapy, more extensive surgery and less necrosis being favourable.

When size was included as a possible prognostic variable only 33 patients were available for analysis. However, in this subgroup it was the most important independent prognostic factor ($P = 0.0011$), patients with smaller tumours having a better prognosis. Also included in the model were necrosis ($P = 0.0026$) and cellularity ($P = 0.0203$), with less necrosis and more cellularity being favourable.

*Deep tumours*

There were 31 patients with tumours arising in the retroperitoneum or the thorax. For the analysis histological type was recorded as leiomyosarcoma, liposarcoma or other. The independent prognostic factors identified were histological type ($P < 0.0001$), sex ($P = 0.0003$), extent of surgery ($P = 0.0168$) and radiotherapy ($P = 0.0237$) with cellularity ($P = 0.0530$) and age ($P = 0.0687$) just failing to reach the conventional level of statistical significance. Liposarcoma emerged as the most favourable histological type for deep tumours, followed by leiomyosarcoma and then the rest. Females had a better prognosis than males and patients having more extensive surgery and/or radical radiotherapy did better.

### Discussion

This study was undertaken to review our experience of the treatment of patients with STS, to analyse the prognostic significance of histological and clinical factors and to compare these with other published results. As the Oncology Department is a specialised cancer treatment unit there may be a bias of referral. Thus patients with larger lesions, possibly difficult to resect, and those with recurrent or metastatic disease may make up a considerable proportion of the population referred. The results demonstrate that several histological and clinical parameters affect prognosis.

Reappraisal of pre-review histological diagnoses is essential since sarcomata are rare and constitute a recognised area of diagnostic difficulty for the general histopathologist. The fact that 17 tumours were recategorised as benign soft tissue lesions (9) and non-sarcomatous malignancies (8) justifies our histological review. Inclusion of these cases would have biased the survival analysis.

The significance of grade has been demonstrated more often than have other histological parameters but in this series is not an independent prognostic factor. However, the independent significance of two of the STS grading criteria, necrosis and mitotic activity, indirectly emphasise the importance of histological grade and suggest that necrosis and mitotic activity may be the most important factors in assessing grade. These two factors seem to be related to the tumour proliferative activity which can be assessed by staining frozen sections of tumours with the monoclonal antibody Ki-67. Initial investigations by Ueda *et al.* (1989) indicate a significant correlation between survival and tumour reactivity for Ki-67 which might be used as one of the histological factors for grading STS.

Costa *et al.* (1984) demonstrated the effect of extent of necrosis on survival and used it in their grading system. Since then other authors (Mandard *et al.*, 1989; Rooser *et al.*, 1988; Trojani *et al.*, 1984) have, like us, shown it to be an independent prognostic factor. Assessment of the extent of necrosis is the most subjective and difficult of the criteria used in grading (Costa *et al.*, 1984; Trojani *et al.*, 1984). A systematic approach to STS, including assessment of necrosis should be developed by all pathologists.

There have been conflicting reports regarding the prognostic significance of size and site. Since there is always a degree of tissue reaction around the tumour the clinical size is not an accurate reflection of tumour volume. Pathological size is probably best used in analysis. The prognostic significance of overall size (Mandard *et al.*, 1989; Ueda *et al.*, 1988) and site (Collin *et al.*, 1987; Markhede *et al.*, 1982) is well documented in the literature. In this study, size was an independent prognostic factor in the superficial STS but not in either the overall group or the deep STS. As expected, due to the surgical problems and, probably, the nature of such tumours, survival was poor for patients with retroperitoneal disease (Bramwell *et al.*, 1985; Collin *et al.*, 1987; Markhede *et al.*, 1982; Tsujimoto *et al.*, 1988). Since retroperitoneal STS tend to be larger at presentation it is difficult to separate size from site as an independent prognostic factor. In this study, few deep STS were completely resected which meant that their pathological size was unknown and could not be included in the proportional hazards model.

In this review the UICC staging, a modified version of the AJC staging, was used. It proved to be of prognostic value only on univariate analysis.

The prognostic significance of symptoms has been reported by Collin *et al.* (1987) and Ueda *et al.* (1988). Heise *et al.* (1986) found a higher recurrence-free survival in patients presenting with mass as compared with other symptoms. The wide range of duration of symptoms has been shown by others (Bramwell *et al.*, 1985; Ueda *et al.*, 1988) and probably relates to the fact that many patients ignore any swelling not accompanied by pain. In our series there was an association of symptoms with site and they did not prove to be of prognostic significance.

The prognostic significance of sex, age and local recurrence has also been reported by one or more investigators (Bramwell *et al.*, 1985; Collin *et al.*, 1987; Markhede *et al.*, 1982; Pinedo *et al.*, 1984; Tsujimoto *et al.*, 1988). Only sex was of prognostic importance in our series.

The mode of treatment affects survival (Dewar & Duncan, 1985). In our series radical surgical treatment and radiotherapy were associated with better survival. There have been many reports on the role of surgical excision alone or followed by adjuvant radiotherapy (Abbas *et al.*, 1981; Coe *et al.*, 1981; Dewar & Duncan, 1985; Markhede *et al.*, 1982; Ueda *et al.*, 1988). A poor survival in patients with unresectable tumours, irrespective of further treatment has been shown by Suit *et al.* (1985) and Gerner *et al.* (1975). Various methods of surgical treatment seem to relate to different rates of local recurrence (Markhede *et al.*, 1982; Shieber *et al.*, 1961; Shiu *et al.*, 1975); this may be simply a reflection of the inadequacy of surgical excision (Abbas *et al.*, 1981; Bell *et al.*, 1989; Collin *et al.*, 1987; Mandard *et al.*, 1989). Radical local excision as described by Simon and Enneking (1976) resulted in a local control rate of 98% in their series of 46 patients. Such extensive surgical approaches frequently involve considerable mutilation and in many cases may be technically impossible. Although initially suggested by Cade (1951) the role of radiotherapy has only relatively recently been established for local control of STS. By treating 64 patients with local (radical) radiotherapy following simple excision of the tumour Suit *et al.* (1975) reported a local control rate of 90.6%. Subsequently Rosenberg *et al.* (1982) stressed the effectiveness of local radiotherapy for local control, avoiding mutilating surgery. In a recent report (Suit *et al.*, 1985) comparing the pooled data of various institutions, the local failure rate was 18.1% for the patients treated by radical surgery or amputation and 18.3% for the patients treated by conservative surgery and postoperative radiation. In our series these figures are 10% and 22.2% respectively.

The following variables have been reported by one or more authors as being independent prognostic factors: age, sex, symptoms, site, depth, size, tumour borders, differentiation, mitotic activity, tumour necrosis, grade, surgical treatment, adequacy of surgical excision, adjuvant chemotherapy, local recurrence and nodal metastases (Collin *et al.*, 1987; Heis *et al.*, 1986; Mandard *et al.*, 1989; Markhede *et al.*, 1982; Trojani *et al.*, 1984; Tsujimoto *et al.*, 1988; Ueda *et al.*, 1988). Our results are within this wide range of findings. They also show the independent significance of radical radiotherapy as a primary or adjuvant treatment.

In most series STS were defined as superficial or deep depending on their relation to the deep fascia. While these definitions are acceptable for STS of the extremity and trunk, they do not apply to the retroperitoneal and thoracic STS. The results of the multivariate analyses done on the superficial STS are as expected. Smaller tumours are more amenable to adequate surgical treatment and radiotherapy. Extent of necrosis tends to reflect the rate of proliferative growth which is in turn related to survival (Ueda *et al.*, 1989).

In summary our clinical findings are in broad agreement with the literature. Our survival analyses reaffirm the importance of radical treatment. They also demonstrate that certain histological features, i.e. necrosis and mitotic activity, are of special significance and they probably relate to assessment of grade more than any of the other factors currently in use. Future studies to determine prognostic factors should include cell kinetics to assess their contribution in predicting biological behaviour and survival. Although the number of cases in this study is not small the subgroups are and we believe that there is a need for further larger co-operative, studies to standardise assessment of grade and to identify prognostic factors. Only by such collaboration can we develop a better clinicopathological understanding of soft tissue sarcomata.

We thank our clinical and pathology colleagues in the Departments of Clinical Oncology and Pathology, University of Edinburgh, for allowing access to the notes and histology material of the patients included in this review. We also thank Dr A. Lessells, Western General Hospital, Edinburgh, Dr P. Walker, Fife Area Laboratory, Kirkcaldy, Drs I. Gibson and A. Lufty, Dumfries and Galloway Royal Infirmary, Drs G. Sclare and R. Davie, Bangour General Hospital, Professor H. Simpson, Glasgow Royal Infirmary, and Professor F. Walker, University of Aberdeen, for providing histology material. Our thanks to the technical and the secretarial staff of the above Laboratories and Departments for their immense help. Finally we thank Miss Jacqueline Kerr for typing the manuscript.

### References

ABBAS, J.S., HOLYOKE, E., MOORE, R. & KARAKOUSIS, C.P. (1981). The surgical treatment and outcome of soft tissue sarcoma. *Arch. Surg.*, **116**, 765.

BELL, R.S., O'SULLIVAN, B., POWELL, J. & 6 others (1989). The surgical margin in soft tissue sarcoma. *J. Bone Joint Surg.*, **71A**, 370.

BRAMWELL, V.H.C., CROWTHER, D., DEAKIN, D.P., SWINDELL, R. & HARRIS, M. (1985). Combined modality management of local and disseminated adult soft tissue sarcomas: a review of 257 cases seen over 10 years at the Christie Hospital and Holt Radium Institute, Manchester. *Br. J. Cancer*, **51**, 301.

CADE, S. (1951). Soft tissue tumours: their natural history and treatment. *Proc. R. Soc. Med.*, **44**, 19.

COE, M.A., MADDEN, F.J. & MOULD, R.F. (1981). The role of radiotherapy in the treatment of soft tissue sarcoma: a retrospective study 1958–1973. *Clin. Radiol.*, **32**, 47.

COLLIN, C., GODBOLD, J., HAJDU, S. & BERNNAN, M. (1987). Localized extremity soft tissue sarcoma: an analysis of factors affecting survival. *J. Clin. Oncol.*, **5**, 601.

COSTA, J., WESLEY, R.A., GLATSTEIN, E. & ROSENBERG, S.A. (1984). The grading of soft tissue sarcomas. Results of a clinicopathologic correlation in a series of 163 cases. *Cancer*, **53**, 530.

COX, D.R. (1972). Regression models and life-tables. *J. R. Stat. Soc., Series B*, **34**, 187.

DEWAR, J.A. & DUNCAN, W. (1985). A retrospective study of the role of radiotherapy in the treatment of soft tissue sarcoma. *Clin. Radiol.*, **36**, 629.

ENZINGER, F.M. & WEISS, S.M. (1988). *Soft Tissue Tumours*. C.V. Mosby: St Louis.

GERNER, R.E., MOORE, G.E. & PICKREN, J.W. (1975). Soft tissue sarcomas. *Ann. Surg.*, **181**, 803.

HEISE, H.W., MYERS, M.H., RUSSELL, W.D. & 7 others (1986). Recurrence-free survival time for surgically treated soft tissue sarcoma patients. Multivariate analysis of five prognostic factors. *Cancer*, **57**, 172.

MANDARD, A.M., PETIOT, J.F., MARNAY, J. & 9 others (1989). Prognostic factors in soft tissue sarcomas. A multivariate analysis of 109 cases. *Cancer*, **63**, 1437.

MARKHEDE, G., ANGERVALL, L. & STENER, B. (1982). A multivariate analysis of the prognosis after surgical treatment of malignant soft tissue tumours. *Cancer*, **49**, 1721.

PINEDO, H.M., BRAMWELL, V.H.C., MOURIDSEN, H.T. & 11 others (1984). Cyvadic in advanced soft tissue sarcoma: a randomised study comparing two schedules. A study of the EORTC soft tissue and bone sarcoma group. *Cancer*, **53**, 1825.

ROOSER, B.O., ATTEWELL, R., BERG, N.O. & RYDHOLM, A. (1987). Prognostication in soft tissue sarcoma. A model with four risk factors. *Cancer*, **61**, 817.

ROSENBERG, S.A., TEPPER, J., GLATSTEIN, E. & 8 others (1982). The treatment of soft tissue sarcomas of the extremities. Prospective randomised evaluation of (1) limb-sparing surgery plus radiation therapy compared with amputation and (2) the role of adjuvant chemotherapy. *Ann. Surg.*, **196**, 305.

RUSSELL, W.D., COHEN, J., ENZINGER, F. & 7 others (1977). A clinical and pathological staging system for soft tissue sarcomas. *Cancer*, **40**, 1562.

SHIEBER, W. & GRAHAM, P. (1961). An experience with sarcomas of the soft tissues in adults. *Surgery*, **52**, 295.

SHIU, M.H., CASTRO, E.B., HAJDU, S.I. & FORTNER, J.G. (1975). Surgical treatment of 297 soft tissue sarcomas of lower extremity. *Ann Surg.*, **182**, 597.

SIMON, M.A. & ENNEKING, W.F. (1976). The management of soft tissue sarcomas of the extremities. *J. Bone Joint Surg.*, **58-A**, 317.

SUIT, H.D., MANIN, H.J., WOOD, W.G. & PROPPE, K.H. (1985). Preoperative, intraoperative and postoperative radiation in the treatment of primary soft tissue sarcoma. *Cancer*, **55**, 2659.

SUIT, H.D., RUSSELL, W.D. & MARTIN, R.G. (1975). Sarcoma of soft tissue: clinical and histopathological parameters and response to treatment. *Cancer*, **35**, 1478.

TROJANI, M., CONTESSO, G., COINDRE, J.M. & 7 others (1984). Soft tissue sarcomas of adults: study of pathological prognostic variables and definition of a histopathological grading system. *Int. J. Cancer*, **33**, 37.

TSUJIMOTO, M., AOZASA, K., VEDA, T., MORIMURA, Y., KOMATSUBARA, Y. & DOI, T. (1988). Multivariate analysis for histological prognostic factors in soft tissue sarcomas. *Cancer*, **62**, 994.

UICC (1987). *TNM Classification of Malignant Tumours*, 4th edition. Springer Verlag: Berlin.

UEDA, T., AOZASA, K., TSUJIMOTO, M. & 4 others (1988). Multivariate analysis for clinical prognostic factors in 163 patients with soft tissue sarcomas. *Cancer*, **62**, 1444.

UEDA, T., AOZASA, K., TSUJIMOTO, M. & 5 others (1989). Prognostic significance of Ki-67 reactivity in soft tissue sarcomas. *Cancer*, **63**, 1607.